# P-1370. Molecular Bases and Mechanistic Insights of NDM-mediated Resistance to Cefiderocol

**DOI:** 10.1093/ofid/ofae631.1547

**Published:** 2025-01-29

**Authors:** Alejandro J Vila, Brenda A Warecki, Maria F Mojica, Pablo Tomatis, Christopher Bethel, Magdalena Rodriguez, Daisuke Ono, Guillermo Bahr, Salvador I Drusin, Diego Moreno, Graciela Mahler, Robert A Bonomo

**Affiliations:** Instituto de Biología Molecular y Celular de Rosario (IBR), Rosario, Santa Fe, Argentina; IBR-CONICET, Rosario, Santa Fe, Argentina; Case Western Reserve University, Cleveland, Ohio; IBR - University of Rosario, Rosario (Argentina), Santa Fe, Argentina; Louis Stokes Cleveland VA Medical Center, Cleveland, Ohio; Facultad de Química, Udelar, Montevideo, Montevideo, Uruguay; Case Western Reserve University, Cleveland, Ohio; Terragene S.A., Rosario, Santa Fe, Argentina; Instituto de Química Rosrio (IQUIR-CONICET), Rosario, Santa Fe, Argentina; IQUIR, Instituto de Química de Rosario, CONICET, Universidad Nacional de Rosario, Rosario, Santa Fe, Argentina; Universidad de la Republica, Montevideo, Montevideo, Uruguay; Case Western Reserve University/ Louis Stokes Cleveland VA Medical Center, Cleveland, OH

## Abstract

**Background:**

Cefiderocol (FDC) is a novel cephalosporin with a siderophore-mimicking moiety that favors uptake into the periplasm through iron transporters. FDC was reported as the only cephalosporin refractory to hydrolysis by metallo-β-Lactamases (MBLs). However, resistance to FDC has been reported due to substitutions in the iron transporter coupled to the expression of the MBLs NDM-1 and NDM-5. In contrast, resistance emergence involving other MBLs such as VIM or IMP are not known. We aimed to identify the interaction of FDC with NDM and other MBLs.

**Table 1.**
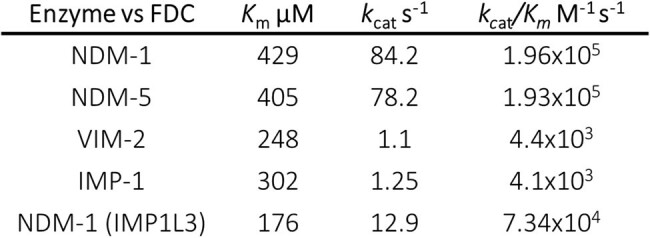
Kinetic parameters of FDC hydrolysis by different MBLs NDM variants show considerable activity in FDC hydrolysis. In contrast, VIM-2 and IMP-1 show poor activity.

**Methods:**

MICs values of FDC against *Escherichia coli* DH10B cells expressing different MBLs cloned into a pHSG298 vector were determined by the microdilution method in iron-depleted cation-adjusted Mueller-Hinton broth. NDM-1, NDM-5, IMP-1 and VIM-2 were expressed in *E. coli* BL21(DE3) pLysS and purified by affinity chromatography. Steady state catalytic parameters were determined by initial rate measurements measured in HEPES buffer, pH 7.5 at 25°C. NMR spectra were run in a 700 MHz Bruker spectrometer. Stopped-flow data were acquired in an Applied Photophysics spectrophotometer.

Mechanism of MBL-mediated resistance to FDC

**Figure:**
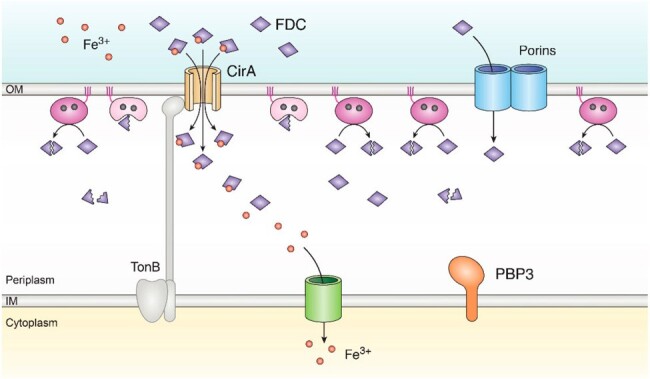

Under high levels of NDM expression, the enzymatic activity counter the cefiderocol action, confering bacterial resistance against this antibiotic.

**Results:**

MIC values of FDC in *E. coli* cells expressing NDM variants were 2 μg/ml, similar to NDM-1. Steady-state kinetic measurements showed that NDM-1 and NDM-5 hydrolyze FDC efficiently, with *k*_cat_/K_M_ values of 1.9 and 1.2 x10^6 M^-1^s^-1^, respectively. In contrast, IMP-1 and VIM-2 displayed poor catalytic efficiencies (Table 1). Electrospray ionization mass spectrometry (ESI-MS) revealed that FDC forms an adduct with MBLs, which is significantly more stable in the case of IMP-1 and VIM-2. Stopped flow experiments under pre-steady state conditions show that both IMP-1 and VIM-2 form a stable adduct with FDC, that results in enzyme inhibition. Instead, NDM-1 is poorly inhibited since the formed adduct is less stable. NMR experiments confirmed the formation of an hydrolysis product and adduct.

**Conclusion:**

Our results indicate that NDM-1 and NDM-5 hydrolyze FDC efficiently, in contrast to the poor activity of IMP-1 and VIM-2. These findings support the observation that clinical resistance is linked to overexpression of NDM variants and not to IMP-1 and VIM-2. This may guide the use of FDC against a specific group of MBLs and justifies pairing with an MBL inhibitor.

**Disclosures:**

**All Authors**: No reported disclosures

